# Exposure to *Brucella* Species, *Coxiella burnetii*, and *Trichinella* Species in Recently Imported Camels from Sudan to Egypt: Possible Threats to Animal and Human Health

**DOI:** 10.3390/pathogens13020179

**Published:** 2024-02-16

**Authors:** Ragab M. Fereig, Amira M. Mazeed, Ashraf A. Abd El Tawab, Mohamed El-Diasty, Ahmed Elsayed, Raafat M. Shaapan, Abdelbaset E. Abdelbaset, Caroline F. Frey, Bader S. Alawfi, Sarah A. Altwaim, Azzah S. Alharbi, Gamal Wareth

**Affiliations:** 1 Division of Internal Medicine, Department of Animal Medicine, Faculty of Veterinary Medicine, South Valley University, Qena 83523, Egypt; 2 Department of Infectious Diseases, Faculty of Veterinary Medicine, Arish University, Arish 45516, Egypt; amera.mohamed@vet.aru.edu.eg; 3 Department of Bacteriology, Immunology, and Mycology, Faculty of Veterinary Medicine, Benha University, Toukh 13736, Egypt; ashraf.awad@fvtm.bu.edu.eg; 4 Agricultural Research Center (ARC), Animal Health Research Institute-Mansoura Provincial Laboratory, (AHRI-Mansoura), Giza 12618, Egypt; dr_mesbah_m@yahoo.com; 5 Agricultural Research Center (ARC), Animal Health Research Institute-Al Shalateen Provincial Laboratory (AHRI-Al Shalateen), Giza 12618, Egypt; dr_ahmed_elsayed_2106@yahoo.com; 6 Department of Zoonoses, National Research Centre, 33 El-Tahrir Street, Dokki 12622, Egypt; rmshaapan2005@yahoo.com; 7 Laboratory of Parasitology, Graduate School of Infectious Diseases, Faculty of Veterinary Medicine, Hokkaido University, Sapporo 060-0808, Japan; a.eweda@aun.edu.eg; 8 Clinical Laboratory Diagnosis, Department of Animal Medicine, Faculty of Veterinary Medicine, Assiut University, Assiut 71526, Egypt; 9 Institute of Parasitology, Department of Infectious Diseases and Pathobiology, Vetsuisse-Faculty, University of Bern, Länggassstrasse 122, CH-3012 Bern, Switzerland; caroline.frey@unibe.ch; 10 Department of Clinical Laboratory Sciences, College of Applied Medical Sciences, Taibah University, Madinah 42353, Saudi Arabia; bawfi@taibahu.edu.sa; 11 Department of Clinical Microbiology and Immunology, Faculty of Medicine, King Abdulaziz University, Jeddah 21589, Saudi Arabiaasalharbi3@kau.edu.sa (A.S.A.); 12 Special Infectious Agents Unit, King Fahd Medical Research Center, King Abdulaziz University, Jeddah 22252, Saudi Arabia; 13 Institute of Bacterial Infections and Zoonoses, Fredrich-Loeffler-Institut (FLI), 07743 Jena, Germany; 14 Institute of Infectious Diseases and Infection Control, Jena University Hospital, 07747 Jena, Germany

**Keywords:** brucellosis, camel, dromedary, serology, iELISA, Egypt

## Abstract

Brucellosis and coxiellosis/Q fever are bacterial infections caused by *Brucella* species and *Coxiella burnetii*, respectively; camels are highly susceptible to both pathogens. Trichinellosis is a parasitic infection caused by various *Trichinella* nematode species. Reportedly, camels are susceptible to experimental infection with *Trichinella* spp., but information on this potential host species is scarce. All three infections are of zoonotic nature and thus of great public health concern. The current study aimed to determine antibodies against the three pathogens in recently imported camels (n = 491) from Sudan at the two main ports for the entrance of camels into southern Egypt using commercial indirect ELISAs. Samples were collected in two sampling periods. The seropositivity rates of *Brucella* spp., *C. burnetii*, and *Trichinella* spp. were 3.5%, 4.3%, and 2.4%, respectively. Mixed seropositivity was found in 1% for *Brucella* spp. and *C. burnetii*. Marked differences were found between the two study sites and the two sampling periods for *Brucella*. A higher rate of seropositivity was recorded in the Red Sea/older samples that were collected between 2015 and 2016 (4.3%, 17/391; odds ratio = 9.4; *p* < 0.030) than in those collected in Aswan/recent samples that were collected between 2018 and 2021 (0/100). Concerning *C. burnetii*, samples collected during November and December 2015 had a significantly higher positivity rate than the other samples (13%, 13/100; OD = 4.8; *p* < 0.016). The same effect was observed for antibodies to *Trichinella* spp., with samples collected during November and December 2015 showing a higher positivity rate than the other samples (7%, 7/100; OD = 10.9; *p* < 0.001). This study provides valuable information on the seroprevalence of *Brucella* spp. and additional novel information on *C. burnetii* and *Trichinella* spp. in recently imported camels kept in quarantine before delivery to other Egyptian regions. This knowledge can be utilized to reduce health hazards and financial burdens attributable to brucellosis, Q fever, and trichinellosis in animals and humans in Egypt.

## 1. Introduction

Camel is an important source of meat and milk, which raises the risks of zoonotic infections spread through food [1,2]. Additionally, due to the potential for transmission to humans through contact with infected dairy products and animals, a high endemic incidence of zoonoses constitutes a substantial threat to human health [3]. Livestock can be infected by various parasitic, bacterial, fungal, and viral zoonoses, among them brucellosis, Q fever, and trichinellosis.

Brucellosis, caused by *Brucella* spp., is a serious bacterial disease causing major public health and economic concerns [4]. While being widespread worldwide, brucellosis is more common in countries with weak public and animal health programs [5]. Macrophages, dendritic cells, placental trophoblasts, and epithelial cells can all be invaded by *Brucella*, a tiny, Gram-negative, non-motile, non-spore-forming, aerobic, intracellular coccobacilli [5,6,7].

Camel brucellosis can cause a major loss in productivity at the herd level by delaying sexual maturity, lengthening calving intervals, and reducing milk production. Diagnosis of brucellosis in male camels is challenging due to non-obvious clinical signs, despite it being seropositive [8]. The main animal reservoirs for *Brucella* are cattle, goats, swine, and sheep, which are infected by *B. abortus*, *B. melitensis*, *B. suis*, and *B. ovis*, respectively. Nearly all regions with camel farming systems, except Australia, have reported camel infections with *B. melitensis* and *B. abortus* [9]. Camels can be infected by different biovars of any of the two species, *B. abortus* [10] and *B. melitensis* [11]. Brucellae could be easily recovered from lymph nodes, vaginal swabs, testicles, and the stomach contents of aborted foetuses; however, isolation from camel milk was uncommon [12].

Q fever (Coxiellosis) is a neglected zoonotic disease caused by the bacterium *Coxiella* (*C*.) *burnetii*. It circulates in wildlife and domestic ruminants. The infection process can be initiated with a very small number of microorganisms. Exposure to domestic animals or their products that test positive for *C. burnetii* typically results in human infection via the aerosol route [13].

Trichinellosis is one of the most common parasitic zoonoses in the world, caused by infection with nematodes belonging to the *Trichinella* genus [14]. *Trichinella spiralis* is a common global parasite that affects a wide range of mammals, including humans. Human trichinellosis has been mainly associated with meat from domestic pigs or wild boars, but other carnivorous and omnivorous animals, and even herbivorous domestic livestock, have been identified as sources of human infections [15,16]. In 1977, *Camelus* sp. was named as a host of *T. spiralis* in India [17]. In Lower Saxony, Germany, eight people had eaten from illicitly imported dried “camel” meat from Egypt, and seven of them subsequently developed strong signs of trichinellosis, which was confirmed by muscle biopsy [18]. The true nature of the meat, however, was never confirmed. Nevertheless, one of the co-authors succeeded in experimentally infecting a camel with >100,000 *Trichinella* sp. L1 obtained from pork meat, and the camel developed numerous encapsulated muscle larvae [18]. Therefore, the potential susceptibility of camels to *Trichinella* spp. infection, and the role they might play for human infection, needs to be further investigated.

In Egypt, several studies have investigated the prevalence of *Brucella* spp. among different animal species, particularly in northern regions [19,20]. *B. melitensis* was previously isolated from cattle, buffaloes, sheep, goats, and Nile catfish [19,21,22], while *B. abortus* was isolated mainly from cattle and buffaloes [12,23,24]. In our recent study, we observed an elevated seroprevalence of *Brucella* species in sheep and goats with a history of abortion when compared to other prevalent abortifacient protozoan agents, e.g., *Toxoplasma gondii* and *Neospora caninum*, using indirect enzyme-linked immunosorbent assay (iELISA) [25]. Numerous reports have consistently investigated the prevalence of *C. burnetii* among camels in Egypt via serological or molecular tools. The seropositivity rates have ranged from 4.5% to 66% in camel samples collected from various regions of Egypt [13,26,27,28]. However, it is important to note that data about the serological or molecular detection of *Brucella* spp. or *C. burnetii* in camels from Egypt are greatly underestimated, with most studies primarily focusing on camels from northern regions or involving only a limited number of tested camels from certain southern regions. Other reports from neighboring countries revealed the high existence of *Brucella* spp. In Sudan, 5 out of 21 (23.8%) tested camels were seropositive to brucellae (*Brucella melitensis* biovar 3, two camels; *Brucella abortus* biovar 6, three camels) [29]. In a camel population from Libya, the seroprevalence of positive sera was 4.1% for *Brucella* spp. [30]. Consistently, in Saudi Arabia, the overall seroprevalence of brucellosis in tested camels was 8% [31]. Similarly, *C. burnetii* prevalence was recorded in numerous African and Middle East countries. In Kenya, the serum testing revealed 18.6% positive seroprevalence of *C. burnetii* [32]. In Jordan, the examination of dromedary camels demonstrated 49.6% seropositivity to *C. burnetii* antibodies, with evidence of maternally derived immunity in calves ≤6 months old [33]. More details on the occurrence and distribution of various zoonotic infections directly related to camels, including brucellosis and coxiellosis in the Middle East region, have been comprehensively reviewed in a recent report [34]. 

In Egypt, *Trichinella* spp. were mainly recorded in pigs, either directly in meat or via antibodies detection in serum. Various reports have investigated *Trichinella* spp. in slaughtered pigs and reported infection rates from 0% to 6% [35]. Seroprevalence of *Trichinella* spp. in pigs in Egypt was 35.6% using an immunofluorescent antibody test (IFAT) [36] and 1.2% using ELISA [35]. However, *T. spiralis* has also been identified in rodents [37] and in dogs [38] in different Egyptian regions. Additionally in humans, the seroprevalence rate of *T. spiralis* (IgG) was 10% (9/90) [39], and 60.9% (56/92) [40] in the examined humans that were linked to the consumption of pork in Egypt. Similarly to Egypt, there are scarce reports on the existence of *Trichinella* spp., either in camels or other animal species. However, a previous report in Libya using trichinoscope revealed the detection of *Trichinella* larvae in 4 (5.7%) of the hedgehogs (*Erinaceus algirus*) and 2 (10.5%) of the red foxes (*Vulpes vulpes*) [41]. In Ethiopia, 5 out of 20 (25%) of human cases that ingested raw meat of wild boar showed signs of illness, and *Trichinella* positivity was confirmed by deltoid biopsy and finding larvae [42]. 

Thus, we aimed to conduct a comprehensive seroprevalence study of brucellosis, coxiellosis, and trichinellosis on a high number of dromedary camels imported to Egypt from Sudan. These camels were quarantined at the two main quarantine points in southern Egypt, i.e., Shalateen, Red Sea governorate, and Abu Simbel, Aswan governorate. As the first checking point in Egypt, seropositive animals are not permitted to transit into other Egyptian areas. Such a strategic measure can significantly contribute to mitigating health hazards and economic losses associated with brucellosis, coxiellosis, and trichinellosis in Egypt.

## 2. Materials and Methods

### 2.1. Ethical Statement

This study was performed according to standard procedures identified by the Research Bio-Ethics standards, Arish University, North Sinai, Egypt. The study was approved by the Research Ethical Committee at Arish University AR/Vet.01. 

### 2.2. Animal Population and Geographic Locations

**Figure 1:**
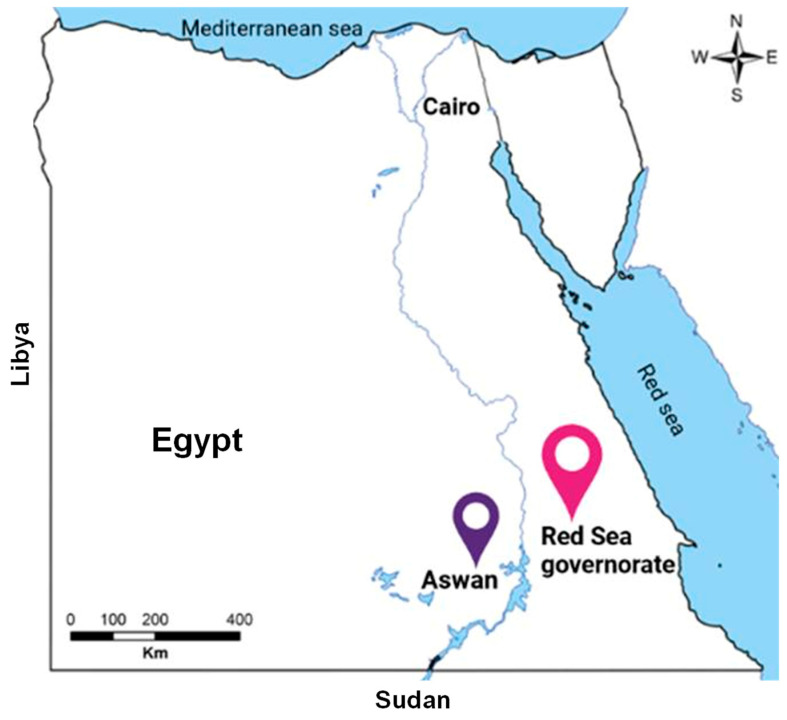
Map of Egypt showing the collection sites of camel sera from southern Egypt. The red symbol refers to the Shalateen quarantine area in the Red Sea governorate, while the bluish symbol indicates the place of Abu Simbel quarantine in the Aswan governorate.

At the two main Egyptian quarantine facilities designated for camels imported from Sudan, a total of 491 blood samples were randomly taken from recently imported camels. No specific criteria were specified for the collected samples in this study. Availability of samples primarily based on the owner’s cooperation and the numbers of recently imported camels at our tested locations determined the current sample populations and groupings. While Abu Simbel quarantine station is in the Aswan governorate and is located in central-south Egypt, Shalateen quarantine station is part of the Red Sea governorate and is located in southeast Egypt (Figure 1). In our prior study, we went into greater detail on the importation, examination, and purpose of the camels utilized in this study [43]. Two different visits—one from November to December 2015 (n = 100 samples) and another from February to March 2016 (n = 291 samples)—were used to collect 391 samples in Shalateen. Additionally, 100 samples were taken at Abu Simbel between September 2018 and March 2021.

### 2.3. Clinical Investigations

Animals underwent clinical examinations before blood sample collection. History of previous diseases or treatment was taken from the animal owners. The examination identified age and gender, as well as the healthy status of the skin, mucous membranes, and general condition. Some examined camels were infested with ticks. However, all of the tested camels were adult (>2 years old) males and showed no visible clinical signs of infections or other health problems.

### 2.4. Serum Sample Collection and Preparation

Blood samples were obtained through jugular vein puncture using glass tubes without anticoagulant agents. Sera were separated from these blood samples, transferred to the South Valley University laboratory (Faculty of Veterinary Medicine, South Valley University, Qena, Egypt), and stored at −20 °C until use for iELISA testing.

### 2.5. Serological Examination of Camel Serum Samples

#### 2.5.1. Rose Bengal Plate Test for Brucellosis Diagnosis

The Rose Bengal Plate test was performed by previously established protocols [44]. The antigen and serum samples were first warmed to room temperature. An equal amount of 30 µL of antigen and sample was pipetted onto one tear-drop area of the brewer diagnostic card in the center using an adjustable micropipette. Both negative and positive control samples were used. Antigen and sample were completely mixed, and the reading was completed after a 4-min rocking interval. As a control, known positive and negative sera were added. Results were recorded as negative or positive according to the absence or presence of agglutination.

#### 2.5.2. Buffered Acidified Plate Antigen Test (BAPAT) for Brucellosis Diagnosis

The test was carried out as described previously [44]. Before testing, the antigen and the serum were warmed to room temperature. A square of glass was covered with 0.08 mL of each serum sample that was being tested. For each serum sample, one drop (0.03 mL) of the antigen was added after it had been completely mixed. The serum and the antigen were thoroughly combined using a sterile spreader. Results were instantly recorded four and eight minutes later. The test contained known control positive and negative sera. Any “flocculating” reaction was seen as a favorable reaction.

#### 2.5.3. iELISA Testing for Brucellosis, Q Fever, and Trichinellosis Diagnosis and Interpretation of Results

Indirect multi-species ELISA for brucellosis (ID.vet, Grabels, France), a confirmatory test for antibody detection of various *Brucella* species, was used to evaluate serum samples for ELISA (*B. abortus*, *B. melitensis*, and *B. suis*). Controls and serum samples were diluted 1:20. For each of the test samples, the measured ODs were used to determine the sample (S) to positive (P) ratio (S/P%) using the following formula:

SP%=OD sample−OD negative controlOD positive control−OD negative control×100

Samples with an S/P% of less than 110% were regarded as negative, those with an S/P% of between 110% and 120% were regarded as doubtful, and those with an S/P% of more than or equal to 120% were regarded as positive. No samples with findings in the doubtful ranges (110–120%) have been noted in the current investigation.

In *C. burnetii* antibody detection, serum samples were analyzed with an indirect multi-species ELISA for Q fever (ID.vet, Grabels, France). Serum samples and controls were diluted 1:50. The ODs obtained were used to calculate the percentage of sample (S) to positive (P) ratio (S/P%) for each of the test samples according to the following formula: S/P (%) = (OD sample − OD negative control)/(OD positive control − OD negative control) × 100. Samples with an S/P% of less than 40% were considered negative; if the S/P% was between 40% and 50%, the result was considered doubtful; those from 50% to 80% were regarded as positive, and it was considered a strong positive if the S/P% was greater than 80%.

Consistently, serum samples were analyzed for *Trichinella* species using an indirect multi-species ELISA for trichinellosis (ID.vet, Grabels, France). Serum samples and controls were diluted 1:20. The ODs obtained were used to determine the percentage of sample (S) to positive (P) ratio (S/P %) for each of the test samples using the following formula: S/P (%) = (OD sample − OD negative control)/(OD positive control − OD negative control) × 100. Samples with an S/P% of less than 50% were regarded as negative, an S/P% between 50% and 60% was regarded as doubtful, and the test was considered positive if the S/P% was greater than or equal to 60%. 

With the use of an Infinite^®^ F50/Robotic ELISA reader (Tecan Group Ltd., Männedorf, Switzerland), the ODs of all ELISA data were read at 450 nm and quantified. Table 1 contains more information on the commercial kits used.

### 2.6. Statistical Analysis

The significance of the differences in the prevalence rates was analyzed with the Fisher exact test, 95% confidence intervals (including continuity correction), and odds ratios using an online statistical website, www.vassarstats.net (accessed on 15 November 2023), as described previously [25,43]. *p*-values and odds ratio were also confirmed with GraphPad Prism version 5 (GraphPad Software Inc., La Jolla, CA, USA). The results were considered significant when the p-value was < 0.05. Group comparisons of % of inhibition were analyzed using one-way ANOVA with Tukey–Kramer post hoc analysis, *p* < 0.05.

## 3. Results

### 3.1. Seroprevalence Rate of Brucella spp., C. burnetii, and Trichinella spp. Antibodies in Camels

**Figure 2:**
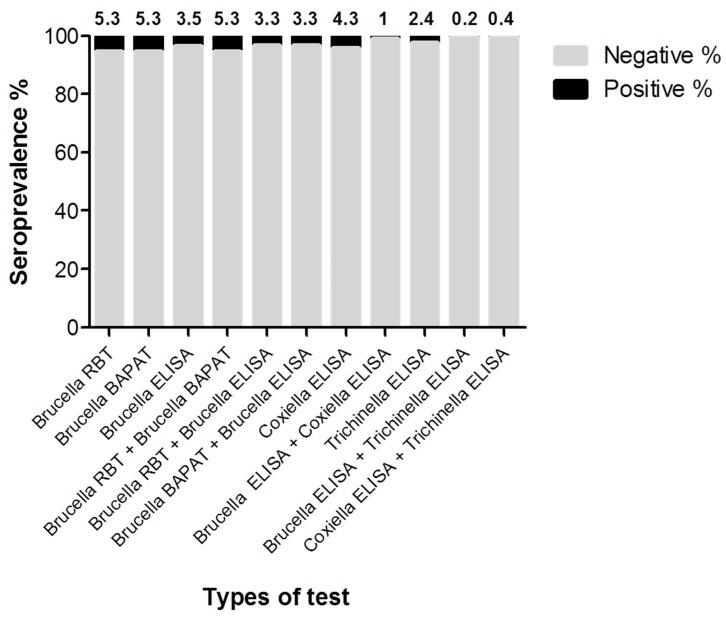
Seroprevalence of *Brucella species*, *Coxiella burnetii*, *Trichinella* spp., and mixed infections in tested camels. Various indirect ELISAs were used for testing the camel (n = 491) as confirmatory tests for detection of specific antibodies against selected pathogens. RBT, Rose Bengal test; BAPAT, Buffered acidified plate antigen test. Values above the bars refer to the estimated seroprevalence rates.

Herein, specific antibodies to *Brucella* species were detected and confirmed in 17 of the 491 surveyed animals (3.5%; 95% CI: 2.1–5.6) using iELISA as a confirmatory test (Figure 2 and Table S1). As a preliminary testing, 26 camel sera were tested positive for *Brucella* species antibodies (5.3%; 95% CI: 3.6–7.8), using RBT and BAPAT as screening tests. Noteworthy, all samples testing positive for RBT were also positive for BAPAT, and similar results have been noticed for seronegative results. Additionally, 16 samples of 491 have shown positive results simultaneously for iELISA (total positive number = 17), RBT, and BAPAT (total positive number = 26) (3.3%; 95% CI: 1.9–5.4). However, iELISA for Brucellosis detected one sample that was negative to RBT and BAPAT.

In addition, a widely used commercial iELISA was used to detect specific antibodies to *C. burnetii* in camel sera. The test revealed 21 camel sera as positive for *C. burnetii* antibodies (4.3%; 95% CI: 2.7–6.6). For *Trichinella*, iELISA testing revealed 12 seropositive cases among all tested camels (2.4%; 95% CI: 1.3–4.4). Analyzing camel sera for mixed infections, antibodies to *Brucella* and *C. burnetii* were demonstrated in five camel samples using only iELISA results as confirmatory tests (1%; 95% CI: 0.4–2.5). In addition, *Trichinella* spp. antibodies were detected in co-infection with *Brucella* spp. in one animal (0.2%; 95% CI: 0.01–1.3) and *C. burnetii* in two animals (0.4%; 95% CI: 0.07–1.6) (Figure 2 and Table S1).

**Figure 3:**
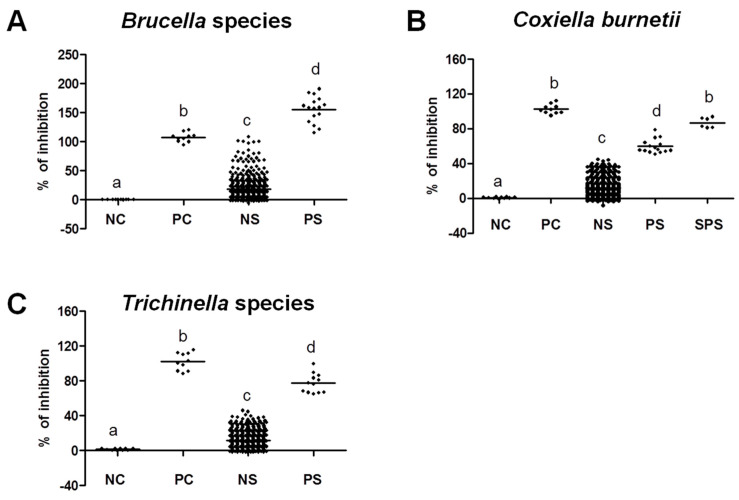
Evaluation of seroreactivity levels among positive and negative samples against selected pathogens. Antibody levels indicated in % of inhibition of positive and negative test samples and control negative and positive samples provided in the kit were compared. (**A**) % of inhibition among field and control samples tested by *Brucella* ELISA kit. (**B**) % of inhibition among field and control samples tested by *Coxiella burnetii* ELISA kit. (**C**) % of inhibition among field samples and control samples tested by *Trichinella* spp. ELISA kit. The different letters above the bars in the graphs indicate the statistically significant differences among groups (one-way ANOVA with Tukey–Kramer post hoc analysis, *p* < 0.05). NC, negative controls; PC, positive controls; NS, negative samples; PS, positive samples; SPS, strong positive samples. Samples were identified as negative, positive, or strong positive based on % of inhibition of the manufacturer’s instructions for each kit.

In addition, group comparisons of percent of inhibition from negative and positive samples and negative and positive controls were assessed to evaluate the seroreactivity against *Brucella* spp., *C. burnetii*, and *Trichinella* spp. (Figure 3). In all of the tested pathogens, the percentage of inhibition representing the antibody levels from the positive samples was significantly higher than that of negative samples and negative controls. This result reflects the ability of our used iELISA kits for efficient discrimination between the seroreactivity levels of tested samples. This effect was indicated in the significant difference among positive and negative samples and also the comparable results between positive samples and positive controls. Given the few assessment trials in camels, this study confirmed the usefulness of all used iELISAs, particularly that of *Trichinella* for the seroprevalence of specific antibodies in camels.

### 3.2. Effects of Collection Place and Timing on Seroprevalence Rate of Brucella spp., C. burnetii, and Trichinella spp. Antibodies in Camels

**Figure 4:**
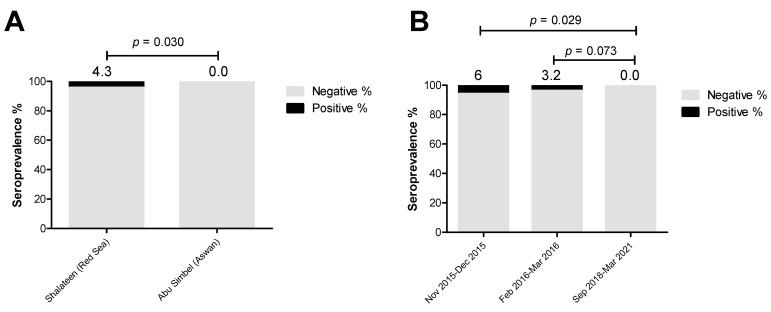
Factors influencing the estimated seroprevalence of *Brucella* species in camels. The effect of collection sites and periods were investigated using univariate logistic regression to identify the risk factors for brucellosis. (**A**) Samples collected at Shalateen (Red Sea governorate, 17/391) showed a significantly higher seropositive rate than those collected at Abu Simbel (Aswan governorate, 0/100). (**B**) In addition, the samples from different collection periods exhibited variable seropositive rates in a statistically significant manner. Compared to samples collected during September 2018–March 2021 (Reference group, 0/100), the seroprevalence was significantly higher in samples collected during November 2015–December 2015 (6/100) and those during February 2016–March 2016 (11/291), but in a non-significant manner. The result is significant at *p* < 0.05, as calculated by Fisher’s exact test. Values above the bars refer to the estimated seroprevalence rates.

On analyzing the influence of the location and period of sample collection as available data on the prevalence of *Brucella* species antibodies in recently imported camels in Egypt, it was evident that both factors significantly impacted the seropositive rate. A significantly higher seroprevalence rate for *Brucella* antibodies was recorded in animals sampled at Shalateen Quarantine, Red Sea governorate (4.3%; odds ratio = 9.4; *p* = 0.030) compared to camel samples collected at Abu Simbel Quarantine, Aswan governorate, where no seropositive sample was detected (Figure 4 and Table S2).

Samples in Shalateen were collected between November and December 2015 and between February and March 2016, and those in Aswan between September 2018 and March 2021. Thus, the same effect was seen when univariable analysis of period of sample collection was performed. Samples collected between November and December 2015 and between February and March 2016 showed higher seropositive rates (6%; OR = 13.8; *p* = 0.029, and 3.8%; OR = 8.2; *p* = 0.073), respectively) than those collected between September 2018 and March 2021 (0.0%), set as a reference group (Figure 4 and Table S2). 

**Figure 5:**
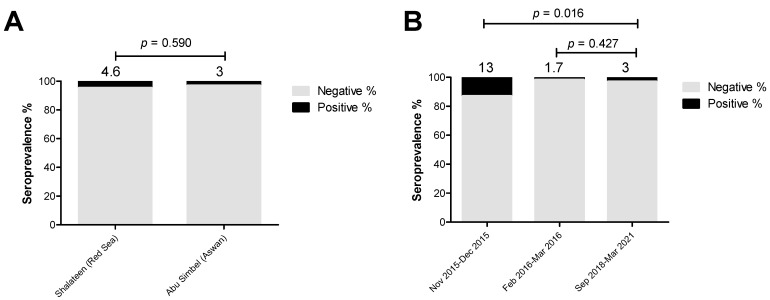
Factors influencing the estimated seroprevalence of *Coxiella burnetii* in camels. The effect of collection sites and periods were investigated using univariate logistic regression to identify the risk factors for brucellosis. (**A**) Samples collected at Shalateen (Red Sea governorate, 18/391) showed a similar seropositive rate to those collected at Abu Simbel (Aswan governorate, 3/100). (**B**) Compared to samples collected during September 2018–March 2021 (Reference group, 3/100), the seroprevalence was significantly higher in samples collected during November 2015–December 2015 (13/100), but it was not different than those collected during February 2016–March 2016 (5/291). The result is significant at *p* < 0.05, as calculated by Fisher’s exact test. Values above the bars refer to the estimated seroprevalence rates.

In the case of analyzing the influence of the location and period of sample collection as available data on the prevalence of *C. burnetii* antibodies in recently imported camels in Egypt, only the collection period affected the seropositive rate significantly. The seroprevalence rate for *C. burnetii* antibodies recorded in animals sampled at Shalateen Quarantine, Red Sea governorate, was similar to those collected at Abu Simbel Quarantine, Aswan governorate (4.6%; OR = 1.6; *p* = 0.590) (Figure 5 and Table S3).

Meanwhile, the seropositive rate of 3% of recently collected samples in Abu Simbel between September 2018 and March 2021 (Reference group) was significantly lower than samples collected in Shalateen between November and December 2015, but was not significantly higher than those collected in Shalateen between February and March 2016 (13%; OR = 4.8; *p* = 0.016, and 1.7%; OR = 0.6; *p* = 0.427, respectively) (Figure 5 and Table S3).

**Figure 6:**
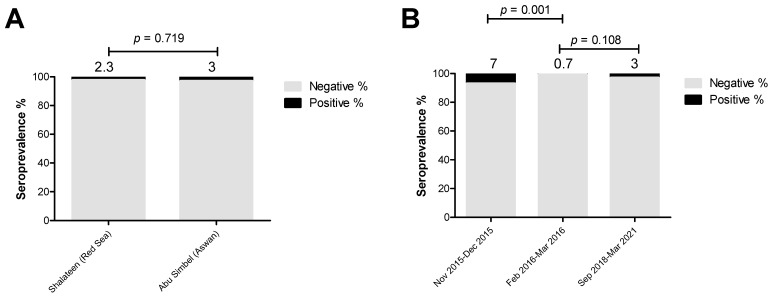
Factors influencing the estimated seroprevalence of *Trichinella* species in camels. The effect of collection sites and periods were investigated using univariate logistic regression to identify the risk factors for brucellosis. (**A**) Samples collected at Shalateen (Red Sea governorate, 9/391) showed a similar seropositive rate to those collected at Abu Simbel (Aswan governorate, 3/100). (**B**) Comparing to samples collected during February 2016–March 2016 (5/291) (Reference group, 2/291), the seroprevalence was significantly higher in samples collected during November 2015–December 2015 (7/100), but it was not different than those collected during September 2018–March 2021 (3/100). The result is significant at *p* < 0.05, as calculated by Fisher’s exact test. Values above the bars refer to the estimated seroprevalence rates.

Concerning *Trichinella* spp. analysis, the collection period affected the seropositive rate significantly. The seroprevalence rate for *Trichinella* spp. antibodies in animals sampled at Shalateen Quarantine, Red Sea governorate (2.3%; OR = 0.8; *p* = 0.719) was similar to those collected at Abu Simbel Quarantine, Aswan governorate (3%; reference group) (Figure 6 and Table S4). The seropositive rate of collected samples in Shalateen Quarantine between November and December 2015 was significantly higher (7%; OR = 10.9; *p* = 0.001) than samples also collected in Shalateen between February and March 2016 (0.7%; reference group), but was not significantly higher than those collected in Abu Simbel Quarantine between September 2018 and March 2021 (3%; OR = 4.5; *p* = 0.108) (Figure 6 and Table S4).

## 4. Discussion

Globally, brucellosis, coxiellosis, and trichinellosis are considered substantial burdens on health and economic sectors. They all cause serious human infections and adversely affect livestock production [4,5,13,45]. Herein, we estimated the seroprevalence of specific antibodies to *Brucella* spp. using various serological tests and *C. burnetii* and *Trichinella* spp. using indirect ELISAs. Although detection of specific antibodies is not a direct evidence for the current infection and expected diseases transmission and infection, it is highly efficient for distinguishing different stages of infections. Except for ELISA of *C. burnetii*, all our used tests have the potential to simultaneously detect IgM and IgG antibodies, the markers for acute and chronic infections, respectively [46,47,48,49]. Even for ELISA of *C. burnetii*, manufacturer-based interpretations of strong positivity might indicate the possible acute infection because of the high correlation between high antibody titer with acute infection [50]. Thus, added to the importance of recognition of epidemiological statuses of such pathogens in camels in Egypt, our findings are alarming for authorities due to the possible potential risk of camel coxiellosis, trichinellosis, and brucellosis transmission in human. Coxiellosis can be transmitted through inhalation of contaminated aerosols. Camels, recognized as potential reservoirs, raise concerns about coxiellosis transmission [51]. Trichinellosis could be transmitted through consumption of raw or undercooked meat from infected animals, leading to gastrointestinal and muscular symptoms. Therefore, camel seropositivity for trichinellosis raises concerns about the potential transmission of the parasite to humans and highlights the importance of safe food handling practices. Brucellosis can be transmitted through contact with infected camels or their products, emphasizing the importance of hygiene and proper cooking. Camel seropositivity warns of potential brucellosis transmission to humans. However, further future studies using molecular approaches are highly encouraged for more accurate evaluation of health hazards. 

In Egypt, despite substantial efforts undertaken by both governmental and non-governmental organizations in the last 30 years, brucellosis remains an ongoing endemic and serious infection. Brucellosis continues to pose a substantial threat to both animal and human health [52]. Camels are not known to be primary hosts of *Brucella*, but they are susceptible to both *B. abortus* and *B. melitensis* [53]. Nevertheless, marked alterations have been reported in numerous laboratory findings, including cytokines (increase in interleukin 1β, IL-10), hematology (normocytic normochromic anemia and lymphopenia), and biochemical variables (hypoproteinemia, hypoalbuminemia, and hypoglycemia) in the *Brucella*-infected rather than the non-infected group [26]. On the contrary, Q fever is usually asymptomatic in animals, but sometimes abortion and reproductive insufficiency can be observed. The bacteria can pass in urine, feces, milk, or birth fluid from the infected animals [13]. 

In Egypt, the seroprevalence of camel brucellosis was recorded in earlier studies as 2–5% in nomadic camels and 8–15% in camels kept under intensive or semi-intensive breeding systems [54]. In addition, 50% of male camels kept in a mixed dairy farm at Fayoum governorate, North Upper Egypt, were seropositive, despite showing no clinical signs [8]. Few studies have focused on detecting the seroprevalence of *Brucella* species in recently imported camels at the two main importing points of camels in Egypt (Shalateen, Red Sea, and Abu Simbel, Aswan). In this regard, the overall seroprevalence rate recorded in the current study was 3.5% via confirmatory iELISA and 5.3% using screening RBT and BAPAT tests. A higher seroprevalence rate of 17.20% was previously recorded in imported Egyptian camels [55] and was 15.5% and 22.8% using RBT and iELISA, respectively [28], and 11.5% and 12.9% via complement fixation test and RBT, respectively [27]. This conflict might be related to the differences in collection times, samples, and methodological approaches. 

In the case of serum antibodies against *C. burnetii*, our demonstrated rate was 4.3% using iELISA. This rate was much lower than previously reported in camels from different regions of Egypt; Klemmer et al., 2018, 40.7% [56], and Selim and Abdel-Fattah, 2020, 22% [57], but similar to Abdallah et al., 2019, 4.6% [58] using serological tests. This difference might be assumed to be the variations in testing location, timing, and animals. The lack of available data hindered the further comparison of our data with other studies.

Regarding *Trichinella* spp. antibodies, low seroprevalence was detected in our study, 2.4%. Notably, the ELISA discriminated very well between seronegative and seropositive animals, building trust in the results. This record was difficult to compare because of lack of data on *Trichinella* spp. in camels worldwide. However, this level of seropositivity was similar to that obtained in our previous report conducted on sera from slaughtered pigs (1.2%) in a Cairo abattoir [35], but lower than that recorded in pigs previously (35.6%; Azab et al., 1988) [36]. Compared to human seroprevalence, our rate was also lower than the 10% obtained previously [39], and the 60.9% recorded in a trichinellosis outbreak [40]. In Egypt, camel meat is a major source of animal protein for a high number of Egyptian people because of its similarity to beef, with lower cost. Moreover, previous reports mentioned the possible transmission of *Trichinella* spp. from camel meat to humans [2,17,18]. Based on these findings, the role of camels in the epidemiology of trichinellosis needs further investigation. This should be a priority, also considering that eating raw camel meat is popular among camel nomads in some regions, and severe foodborne outbreaks have occurred due to this habit. Although the observed differences in seropositive rates of brucellosis in the current study were minimal, they may be attributed to the principals of each used serological test (RBT and BAPAT, detect IgG or IgM; ELISA, detects IgG only). Various factors can contribute to variable seropositive rates among the tested pathogens, such as the pathogens type, host susceptibility, and mode of transmission. 

The utility of this result in an application for mitigating brucellosis risks in Egypt makes it crucial to assess and confirm. The majority, if not all, of the camels we examined in Egypt were adult males since the Sudanese government forbids the export of female camels for use in food. The “Dabuka journey,” which entails a long walk from Sudan to Egyptian border ports, is the typical method for moving dromedaries. Since this journey often takes days or weeks to reach Egypt, Sudan may be suspected as the source of infection. Indeed, numerous reports revealed the high prevalence and endemicity of brucellosis among camels in various Sudanese regions [29,59].

In the two quarantine stations, we discovered clear discrepancies in the seroprevalence of *Brucella* species antibodies. The current investigation found that camels recently imported to the Shalateen quarantine in the Red Sea had a greater seroprevalence of *Brucella* species antibodies (4.3%; 17/391) than those gathered at the Abu Simbel quarantine in Aswan (0/100). Data on the seroprevalence of *Brucella* species are underestimated, which is particularly concerning as these animals from Sudan are mainly destined for human consumption. A similar tendency was also reported in the case of seropositive rate against *C. burnetii* antibodies (Shalateen 4.6% vs. Abu Simbel 3%, although it was not in a significant manner (*p* = 0.590). In the case of *Trichinella* spp., differences in collection place did not affect the seropositive rate in a significant manner. hile, collection time was reported as a predisposing factor for *Trichinella* spp. specific antibodies in tested camels. 

We contend that the distinct origins in Sudan and varied travel paths of the camels in the two quarantine stations played a critical role, even if we cannot completely rule out that these discrepancies were brought on by the different sampling years. Camels traveled far from Eastern Sudan to Southeast Egypt before settling in Shalateen. Moreover, camels from Eastern Sudan are quarantined in the government-run quarantine facility close to Kassala city before being transported to Egypt. Camels owned by nomadic tribes initially came from Western Sudan and entered Egypt through the Abu Simbel region. In Egypt, a previous study has reported an opposite result for *Brucella* spp. seroprevalence than ours (herein, 3.5% in Shalateen vs. 0.0% in Abu Simbel) because a higher prevalence was recorded in Abu Simbel (25.8%) than those recorded in Shalateen (16.9%) using indirect ELISA [28]. In addition, previous studies in Sudan have not shown any bias towards the incidence of brucellosis in the eastern region of Sudan, as noticed in our study [8,29,59]. This might be attributable to insufficient preventive measures and the lack of adequate control programs as well as uncontrolled or illegal animal transportation across borders between Egypt and Sudan [60,61]. Notably, in both cases of *Brucella* spp. and *C. burnetti*, and to a lower extent, *Trichinella* spp., seropositive rates were higher in older samples collected from November 2015 to December 2015 or February 2016 to March 2016 than those collected recently from September 2018 to March 2021. However, these findings can be attributable to the confinement of older samples to the Shalateen region, where higher seropositive rates were also detected for both infections. 

Accordingly, the high differences recorded in the current study among the two investigated areas or different times of sample collection might be attributable the tested population criteria, timing of collection, and variation in sample size among the two places (391 animals from Shalateen versus 100 animals from Abu Simbel). However, more studies focusing on the seroprevalence of *Brucella* spp., *C. burnetii*, and *Trichinella* spp. antibodies in recently imported camels at both quarantine stations are required.

Relying on variations of seroprevalence based on timing or period of sample collection, other studies also reported similar tendencies for our tested pathogens. In the case of brucellosis, numerous reports advocated that when the weather condition favors the increase in the transmission rates of brucellosis in livestock, humans, wild animals, and the environment, the incidence of the disease increases significantly, and vice versa [28,62]. Similar findings were reported in camels from the Shalateen area, our investigated region [61]. In the same context, although *C. burnetii* transmission is mainly airborne, ticks may act as vectors and play an important role in the natural cycle of transmission of coxiellosis among wild vertebrates and livestock, thus seasonal fluctuations are highly expected [57,63,64]. Regarding *Trichinella* infections, seasonal fluctuations were also reported in different regions of the world. Climate changes with increasing temperatures and reduction of environmental humidity significantly altered the biology of both the parasitic larvae and susceptible host species [39,45,65].

Given the induction of reproductive problems of both *Brucella* spp. or *C. burnetii* in camels, our seropositive rates might be an indicator for exposure of the positive camels to such problems. Brucellosis in camels can induce delay in sexual maturity, lengthening calving intervals [9]. In addition, *C. burnetii* sometimes causes abortion and reproductive insufficiency [13]. Because our used camels were mostly males imported for the purpose of meat consumption, we could not investigate the statistical influence of sex on the seropositive rates for any of our tested pathogens. However, our seropositive rate of *Brucella* spp. (3.5–5.3%) in tested male camels was similar to those demonstrated in male camels (7.1%) from the same area of our tested samples [61], and lower than those reported by Khan et al. (2020) in camels from various regions in Egypt (17.6–34.9%) [28]. In the case of *C. burnetii* seroreactvity (4.3%), it was significantly lower than those recorded in male camel sera (17%) collected from different Egyptian regions [57].

There are no vaccine programs dedicated to the control of *C. burnetii* or *Trichinella* spp. in Sudan. In addition, *Brucella* vaccination programs are occasionally applied and mostly given to dairy cows and rarely to dairy camel cows reared in extensive or semi-extensive farms. Meanwhile, our tested camels, mainly consisting of males, were reared in desert or semi-desert areas and were not subjected to any kind of veterinary care unless outbreaks or serious health problems arose. Furthermore, the quarantines in which the recently imported camels were received in Egypt are not concerned with the vaccination of collected camels. Consequently, we assumed that the reported seropositivity rates for all our tested pathogens are genuinely representative of past or recent natural infections.

## 5. Conclusions

The recorded seroprevalence rates of brucellosis, coxiellosis, and trichinellosis among imported camels in the current study highlight the potential risks associated with the introduction of infected camels into Egypt. This study confirmed the previous data on the existence of *Brucella* spp. in camels from our tested areas. However, we presented novel data on *C. burnetii* occurrence in camels of such areas (Shalateen and Abu Simbel quarantines) in Egypt. Consistently, we provided the first serological evidence of *Trichinella* spp. infection in camels worldwide. This required the need for the establishments of tight measures at quarantine portals to prevent the crossover-movement of infected animals. 

## Figures and Tables

**Table 1:** Details of used test kits.

Test	Manufacturer	Antigen	Principal	Sensitivity *	Specificity *	Remarks
RBT	Veterinary Sera and Vaccine Research Institute Abassia, Cairo, Egypt	*Brucella abortus* strain 99 cells stained with Rose Bengal at a concentration of 8% in lactate buffer pH (3.65 ± 0.05)	Positive samples were judged by variable degrees of agglutination. Detects any kind of antibodies (IgG or IgM)	100%	99.89%	Sensitivity and specificity were validated against cattle sera based on Hosein et al., 2021 [23].
BAPAT	Veterinary Sera and Vaccine Research Institute Abassia, Cairo, Egypt	*B. abortus* strain 99 cells stained with a crystal violet brilliant green at a concentration of 11% in lactate buffer (pH 3.7 ± 0.03)	Positive samples were judged by variable degrees of agglutination. Detects any kind of antibodies (IgG or IgM)	100%	99.89%	Sensitivity and specificity were validated against cattle sera based on Hosein et al., 2021 [23].
*Brucella* ELISA	ID.vet Innovative Diagnostics, Grabels, France	LPS of *Brucella* species	Indirect multi-species ELISA using anti-multi-species-IgG-HRP	100% (95% CI: 89.57–100%)	99.74% (95% CI: 99.24–99.91%)	Sensitivity and specificity based on manufacturer data sheet
*Coxiella* ELISA	ID.vet Innovative Diagnostics, Grabels, France	Phase I and II proteins of *C. burnetii*	Indirect multi-species ELISA using anti-multi-species-IgG-HRP	100% (95% CI: 89.57–100%)	99.74% (95% CI: 99.24–99.91%)	Sensitivity and specificity based on manufacturer data sheet
*Trichinella* ELISA ^#^	ID.vet Innovative Diagnostics, Grabels, France	*Trichinella* excreted/secreted antigen	Indirect multi-species ELISA using anti-multi-species IgG or IgM-HRP	90.7% (95% CI: 89.1–92.4)	100% (95% CI: 98.95–100)	Sensitivity and specificity based on manufacturer data sheet

RBT, Rose Bengal test; BAPAT, Buffered Acidified Plate Antigen Test; * The sensitivity and specificity of the diagnostic kits were provided by the manufacturer of the kits; 95% CI, 95% confidence interval; ^#^ Detects antibodies to *T. spiralis*, *T. pseudospiralis*, *T. britovi*, and *T. nativa*.

## Data Availability

All data generated and analyzed during this study are included in this published article. Raw data supporting the findings of this study are available from the corresponding author on request.

## Supplementary Materials

The following supporting information can be downloaded at https://www.mdpi.com/article/10.3390/pathogens13020179/s1, Table S1. Seroprevalence of *Brucella* species, *Coxiella burnetti*, and *Trichinella* species in camels using various serological tests. Table S2. Factors influencing the estimated seroprevalence of *Brucella* species in camels. Table S3. Factors influencing the estimated seroprevalence of *Coxiella burnetii* in camels. Table S4. Factors influencing the estimated seroprevalence of *Trichinella* species in camels.
