# Supplementary material for: Exposure to Brucella Species, Coxiella burnetii, and Trichinella Species in Recently Imported Camels from Sudan to Egypt: Possible Threats to Animal and Human Health
Source: Pathogens. 2024 Feb 16;13(2):179. doi: 10.3390/pathogens13020179 (PMC10892970; doi:10.3390/pathogens13020179)
Supplement: Supplementary file 1 [file pathogens-13-00179-s001.zip › pathogens-2847154-supplementary.pdf]

Table S1. Seroprevalence of *Brucella* species, *Coxiella burnetti*, and *Trichinella* species in camels using various serological tests.

Type of test	No. of tested	No. of Negative (%)	No. of positive (%)	95% CI*
RBT	491	465 (94.7)	26 (5.3)	3.6-7.8
BAPAT	491	465 (94.7)	26 (5.3)	3.6-7.8
Bruc. ELISA	491	474 (96.5)	17 (3.5)	2.1-5.6
RBT+BAPAT	491	465 (94.7)	26 (5.3)	3.6-7.8
RBT+Bruc. ELISA	491	475 (96.7)	16 (3.3)	1.9-5.4
BAPAT+Bruc. ELISA	491	475 (96.7)	16 (3.3)	1.9-5.4
Cox. ELISA	491	470 (95.7%)	21 (4.3)	2.7-6.6
Bruc. ELISA+Cox. ELISA	491	486 (99%)	5 (1)	0.4-2.5
Trich. ELISA	491	479 (97.6)	12 (2.4)	1.3-4.4
Bruc. ELISA+Trich. ELISA	491	490 (99.8)	1 (0.2)	0.01-1.3
Cox. ELISA+Trich. ELISA	491	489 (99.6)	2 (0.4)	0.07-1.6

* 95% CI calculated according to method described by (http://vassarstats.net/).

Table S2. Factors influencing the estimated seroprevalence of *Brucella* species in camels.

Analyzed factor	No. of tested	No. of negative (%)	No. of positive (%)	OR (95% CI)*	*p- value**
**Collection region**					
Shalateen (Red Sea)	391	374 (95.7)	17 (4.3)	9.4 (0.6-157.6)	0.030
Abu Simbel (Aswan)	100	100 (100)	0	Ref	Ref
**Collection time**					
Nov 2015-Dec 2015	100	94 (94)	6 (6)	13.8 (0.8-249)	0.029
Feb 2016-Mar 2016	291	280 (96.2)	11 (3.8)	8.2 (0.5-141.2)	0.073
Sep 2018-Mar 2021	100	100 (100)	0	Ref	Ref

* Odds ratio at 95% confidence interval and *p*- value were calculated by GraphPad Prism version 5. The result is significant at $p < 0.05$ as calculated by Fisher's exact test. Ref.; value used as a reference.

Table S3. Factors influencing the estimated seroprevalence of *Coxiella burnetii* in camels.

Analyzed factor	No. of tested	No. of negative (%)	No. of positive (%)	OR (95% CI)*	*p- value**
**Collection region**					
Shalateen (Red Sea)	391	373 (95.4)	18 (4.6)	1.6 (0.5-5.4)	0.590
Abu Simbel (Aswan)	100	97 (97)	3 (3)	Ref	Ref
**Collection time**					
Nov 2015-Dec 2015	100	87 (87)	13 (13)	4.8 (1.3-17.5)	0.016
Feb 2016-Mar 2016	291	286 (98.3)	5 (1.7)	0.6 (0.1-2.4)	0.427
Sep 2018-Mar 2021	100	97 (97)	3 (3)	Ref	Ref

* Odds ratio at 95% confidence interval and *p*- value were calculated by GraphPad Prism version 5. The result is significant at $p < 0.05$ as calculated by Fisher's exact test. Ref.; value used as a reference.

Table S4. Factors influencing the estimated seroprevalence of *Trichinella* species in camels.

Analyzed factor	No. of tested	No. of negative (%)	No. of positive (%)	OR (95% CI)*	*p- value**
**Collection region**					
Shalateen (Red Sea)	391	373 (97.7)	9 (2.3)	0.8 (0.2-2.9)	0.719
Abu Simbel (Aswan)	100	97 (97)	3 (3)	Ref	Ref
**Collection time**					
Nov 2015-Dec 2015	100	93 (93)	7 (7)	10.9 (2.2-53.3)	0.001
Feb 2016-Mar 2016	291	289 (99.3)	2 (0.7)	Ref	Ref
Sep 2018-Mar 2021	100	97 (97)	3 (3)	4.5 (0.7-27.2)	0.108

* Odds ratio at 95% confidence interval and *p*- value were calculated by GraphPad Prism version 5. The result is significant at $p < 0.05$ as calculated by Fisher's exact test. Ref.; value used as a reference.
